# Protein Corona Hinders N-CQDs Oxidative Potential and Favors Their Application as Nanobiocatalytic System

**DOI:** 10.3390/ijms22158136

**Published:** 2021-07-29

**Authors:** Joanna Czarnecka, Mateusz Kwiatkowski, Marek Wiśniewski, Katarzyna Roszek

**Affiliations:** 1 Department of Biochemistry, Faculty of Biological and Veterinary Sciences, Nicolaus Copernicus University in Toruń, 87-100 Toruń, Poland; 2 Chair of Plant Physiology and Biotechnology, Faculty of Biological and Veterinary Sciences, Nicolaus Copernicus University in Toruń, 87-100 Toruń, Poland; m.kwiatkowski@doktorant.umk.pl; 3 Physicochemistry of Carbon Materials Research Group, Faculty of Chemistry, Nicolaus Copernicus University in Toruń, 87-100 Toruń, Poland

**Keywords:** nitrogen-containing carbon quantum dots, protein corona, enzyme immobilization, cytotoxicity, nanobiocatalytic systems

## Abstract

The oxidative properties of nanomaterials arouse legitimate concerns about oxidative damage in biological systems. On the other hand, the undisputable benefits of nanomaterials promote them for biomedical applications; thus, the strategies to reduce oxidative potential are urgently needed. We aimed at analysis of nitrogen-containing carbon quantum dots (N-CQDs) in terms of their biocompatibility and internalization by different cells. Surprisingly, N-CQD uptake does not contribute to the increased oxidative stress inside cells and lacks cytotoxic influence even at high concentrations, primarily through protein corona formation. We proved experimentally that the protein coating effectively limits the oxidative capacity of N-CQDs. Thus, N-CQDs served as an immobilization support for three different enzymes with the potential to be used as therapeutics. Various kinetic parameters of immobilized enzymes were analyzed. Regardless of the enzyme structure and type of reaction catalyzed, adsorption on the nanocarrier resulted in increased catalytic efficiency. The enzymatic-protein-to-nanomaterial ratio is the pivotal factor determining the course of kinetic parameter changes that can be tailored for enzyme application. We conclude that the above properties of N-CQDs make them an ideal support for enzymatic drugs required for multiple biomedical applications, including personalized medical therapies.

## 1. Introduction

One of the most important features ascribed to carbonaceous nanomaterials, including but not limited to carbon quantum dots (CQDs), is their potential to trigger oxidative stress in the cellular microenvironment [1]. Numerous reports have been published on the oxidative properties of CQDs, reactive oxygen species (ROS) production, and their contribution to oxidative stress conditions [1,2,3]. The question arises, how do the CQD oxidative properties interplay with the cellular microenvironment and/or with the immobilized active molecules? In our opinion, the context of oxidative properties of CQDs in their application as enzyme carriers seems to be an unexploited and not fully recognized issue in the field of nanomedicine. Nevertheless, the most recent literature data suggest that CQDs are promising nanodevices for theranostics (combining drug delivery and imaging), benefiting from their biocompatibility, ability to penetrate the cell membranes and to accumulate in cytoplasm [4,5,6,7]. One of the possible solutions to protect cells from oxidative damage is the protein coat formed on the nanomaterial surface. Several studies were performed to elucidate the protein corona formation on various nanomaterials, including carbon quantum dots, and the decrease in cytotoxicity is commonly attributed to this coating [8,9]. The formation of protein corona can also give rise to undesirable results within biological systems. Protein adsorption influences the size and charge of nanomaterial, alters the biodistribution and internalization, thus reducing the efficacy of, e.g., drug delivery. An in-depth understanding of the issues related with protein coating composition, structure and dynamics in bio-fluids are required [8].

The development of enzyme-based medical applications has been at least as extensive as industrial ones, reflecting the magnitude of potential benefits [10] (and references therein). As enzymes are highly specific biological catalysts, they could make the most desirable therapeutics for personalized treatment. Unfortunately, a number of factors severely reduce this potential utility, e.g., sensitivity to physicochemical conditions, inhibitors, proteases, or structure instability [11]. In contrast to the industrial use of enzymes, therapeutically useful enzymatic proteins are required in relatively tiny amounts but at a very high degree of purity and specificity. Their kinetic properties, such as Michaelis constant value (K_m_) and reaction velocity, are pivotal for obtaining the maximally efficient biocatalyst, active even at negligible enzyme and substrate concentrations.

Enzyme immobilization is one of the most powerful techniques to obtain biocatalytic systems with appropriate properties and extended lifetime [11,12,13,14]. In general, immobilization positively affects the structure of enzymatic proteins, increasing their thermal stability and resistance to pH, solvents, and denaturing agents [15,16,17]. Chemical immobilization of enzymes results in obtaining stable nanobiocatalytic systems; however, it often requires the use of toxic linkers, which excludes this method from some applications, such as in the food industry or biomedicine [18]. Physical immobilization methods are characterized by weaker interactions between carrier and protein: hydrogen bonds, van der Waals or hydrophobic interactions [19]. Physical adsorption is also more attractive because enzymes can be reversibly removed from carriers under mild conditions without losing their enzymatic activity [20].

A large variety of inorganic and organic, as well as hybrid and composite materials may be used as stable and efficient supports for biocatalysts. Carbonaceous nanomaterials are among the most favored supports. They have been developed for many biomedical applications, including immobilization of therapeutics, drug delivery, medical imaging, and antimicrobial coatings [21,22]. Intriguingly, nanomaterials are reported to influence the activity of immobilized active molecules in nanobiocatalytic systems [14,16,22].

Carbon quantum dots meet the majority of numerous requirements for the perfect enzyme support. They are characterized by negligible cytotoxicity in comparison to inorganic quantum dots or other highly graphitized carbonaceous materials, high biocompatibility, and low cost of their synthesis. CQDs are chemically inert, have many functional groups on their surface, are well dispersed in water, and additionally, can be easily decorated [1,4,23,24,25]. A sustainable and pollution-free preparation of biocompatible nitrogen-containing carbon quantum dots (N-CQDs) from biomass waste has recently drawn particular attention [26,27]. Great expectations are connected using N-CQDs in biomedical imaging, diagnostics, and as carriers of drugs, genes or other bioactive molecules. The fluorescent properties of quantum dots allow tracking their distribution in the cells or even within tissues and organs [4,28,29]. Doping CQDs with N atoms is considered to be an efficient strategy to improve their photocatalytic and/or fluorescent properties [27,29]. On the other hand, there are doubts concerning the precise quantification of CQD inside the cells by reason of fluorescence quenching, which has been clarified in recent papers [30,31].

In this work, we aimed at shedding more light on the biochemical properties of nitrogen-containing N-CQDs that can serve for the immobilization of enzymes, particularly those with therapeutic potential. We report that despite the intrinsic N-CQD ability to form ROS, the protein corona interacting with quantum dots surface reduces their oxidative properties while does not quench their fluorescence. Biocompatible N-CQDs are readily internalized by different types of cells cultured in vitro. What is more important, N-CQDs were used as immobilization supports controlling the linked enzyme activity through the enzyme-to-carrier ratio. Catalase (EC 1.11.1.6), β-D-galactosidase (EC 3.2.1.23), and apyrase (EC 3.6.1.5), enzymes varying in structure and properties, were used for the immobilization study, all of them increasing the enzymatic activity and efficiency.

## 2. Results

### N-CQD Influence on Oxidative Stress and Cell Viability

The extensive physicochemical characteristics of gelatin-derived, nitrogen-containing CQDs was published recently [30]. Here, we present a thorough analysis of their oxidative properties, cytocompatibility, cellular uptake, and applicability as enzyme immobilization supports.

**Figure 1:**
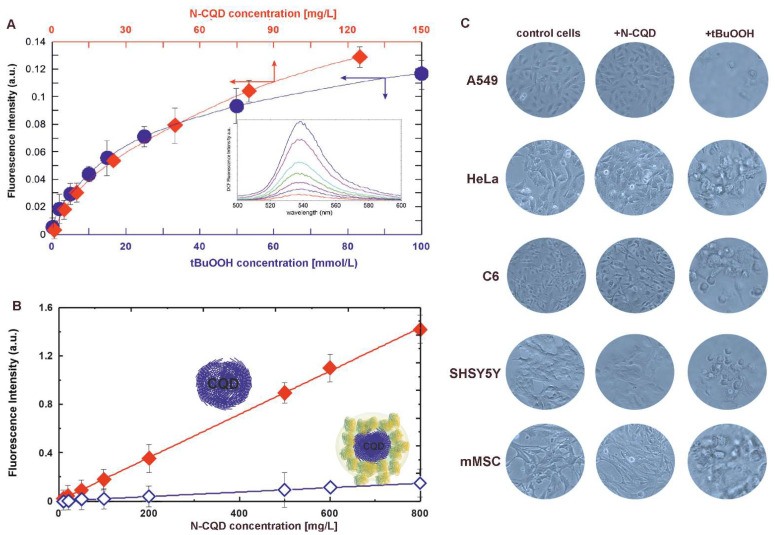
Oxidative properties of N-CQD and tBuOOH—(**A**) fluorescence of dichlorofluorescein (DCF) reflects the amount of ROS produced in the presence of N-CQD (red) and tBuOOH (blue); inset—fluorescence spectra of DCF, (**B**) oxidative properties of N-CQD before (filled diamonds) and after (empty diamonds) albumin coating, (**C**) microscopic images of cells from different cell lines cultured with N-CQDs (20 µg/mL in culture medium) and tBuOOH (10 mM) indicate different oxidative toxicity of both compounds.

To analyze the oxidative potential of N-CQDs and their ability to initiate oxidative stress inside the cells, we have compared the N-CQD-mediated reactive oxygen species (ROS) formation with tert-butyl-peroxide (tBuOOH) as the positive control. The ROS generation has been determined through the ability to oxidize non-fluorescent 2′,7′-dichlorodihydrofluorescein (DCFH_2_) into highly fluorescent DCF. Surprisingly, the amount of N-CQD-derived ROS was comparable with the oxidative properties of millimolar concentrations of tBuOOH—see Figure 1A. Nevertheless, the cell exposure to N-CQDs did not contribute to the oxidative stress conditions (see also Figure 4), cell damage, or death that can be observed in microscopic images—see Figure 1C. Thus, one can conclude that the compared concentrations of N-CQDs (~20 µg/mL in culture medium) and tBuOOH (~10 mM) had a similar ability for ROS formation but considerably different toxicity for cell cultures.

With regard to this data, leading to the conclusion about the potential oxidative nature of CQDs, we further aimed at the elucidation of the phenomena of N-CQD biocompatibility through their bio-interactions with proteins. The 24-hour CQDs incubation, with different ratios of bovine serum albumin to N-CQD, results in protein adsorption on the quantum dot surface, and as a consequence, the formation of the so-called protein corona. Such an albumin coating decreases the N-CQD oxidative properties, reflected with the ability to oxidize DCFH_2_ (Figure 1B).

**Figure 2:**
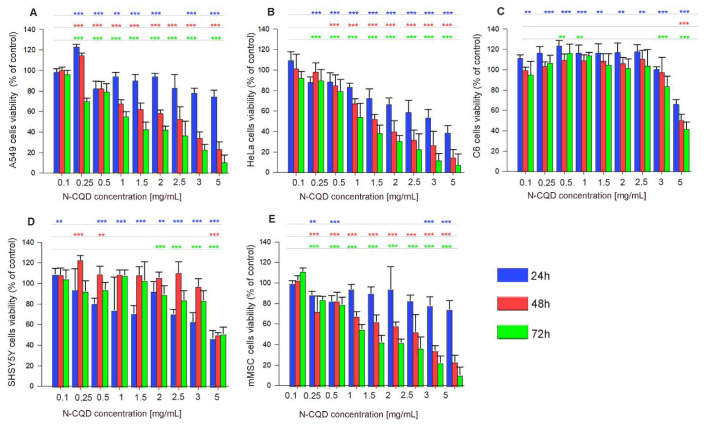
Viability of different cell lines after exposure to growing concentrations of N-CQDs: (**A**) A549, (**B**) HeLa (**C**) C6, (**D**) SH-SY5Y, (**E**) mMSC. All results, including statistical significances, are colored by culture time: 24 h (blue), 48 h (red), and 72 h (green). The statistical significance of the differences is related to control samples and marked on the graphs with asterisks (* for *p* ≤ 0.05, ** for *p* ≤ 0.01, *** for *p* ≤ 0.001).

The N-CQD influence on in vitro cell viability has been studied in a wide range of concentrations (from 100 to 5000 μg/mL) using five distinct cell lines—see Figure 2A–E. Regardless of the broad spectrum of cell types and their properties, increasing N-CQD concentrations caused only a negligible decrease in viability during the first 24 h in human lung epithelial cells A549, rat glioma C6, and mouse mesenchymal stem cells mMSC. In the initial 24 h culture, N-CQD slightly increased the glioma C6 cell viability (Figure 2C). Qualitatively similar results were also obtained for SH-SY5Y cells (Figure 2D); however, a slight decrease in their viability was noticeable in the first 24 h of culture, probably due to a lower proliferation rate.

In general, glioma C6 cells and neuroblastoma SH-SY5Y cells seem to be the most resistant to high concentrations of N-CQD in the cell microenvironment. The EC50 values specifying the material toxicity for all tested cells were only achieved after 72 h of exposure and at concentrations exceeding 1 mg/mL.

**Figure 3:**
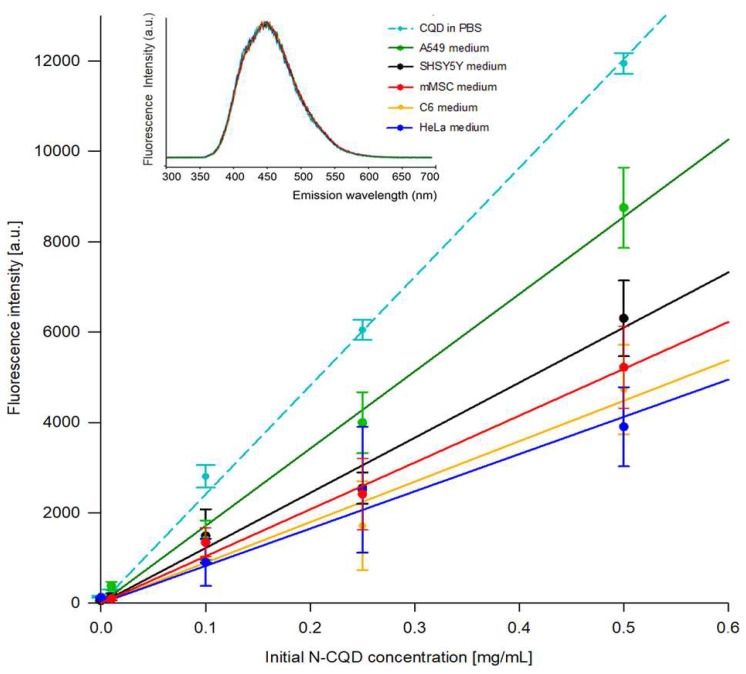
The decrease in N-CQD concentration in cell culture media after 72 h of culture. Inset: fluorescence emission spectra of 50 µg/mL N-CQD in different cell culture media.

The differences in the cell response to N-CQD exposure, suggesting the biocompatibility of studied material, inspired us to determine the amount of nanomaterial internalized by distinct cell types. The quantitative determination of N-CQDs absorbed should be based on the depletion of material concentration in the extracellular environment by reason of fluorescence quenching inside the cells [30]. The quantity of N-CQDs inside different cells was systematically analyzed based on the CQD fluorescence intensity in the culture media collected and compared with control medium (with N-CQD, without cells)—see Figure 3. The results shown in inset in Figure 3 indicate that no components of the culture media changed the carbon quantum dots fluorescence maxima or RFU values. Therefore, the culture media would not affect the possibility of the fluorescence-based determination of N-CQD depletion.

The obtained results clearly indicated variable N-CQDs internalization ability of the tested cell lines. After the total 72-h incubation, glioma C6, SH-SY5Y and mMSC cell lines absorbed similar to HeLa cells quantity of N-CQDs, while the N-CQD internalization rate into A549 cells were considerably lower—see Table 1. It has to be noted that the rate of CQD internalization into different cells is one of the pivotal features defining the nanocarriers and their applicability as drug delivery vehicles.

**Figure 4:**
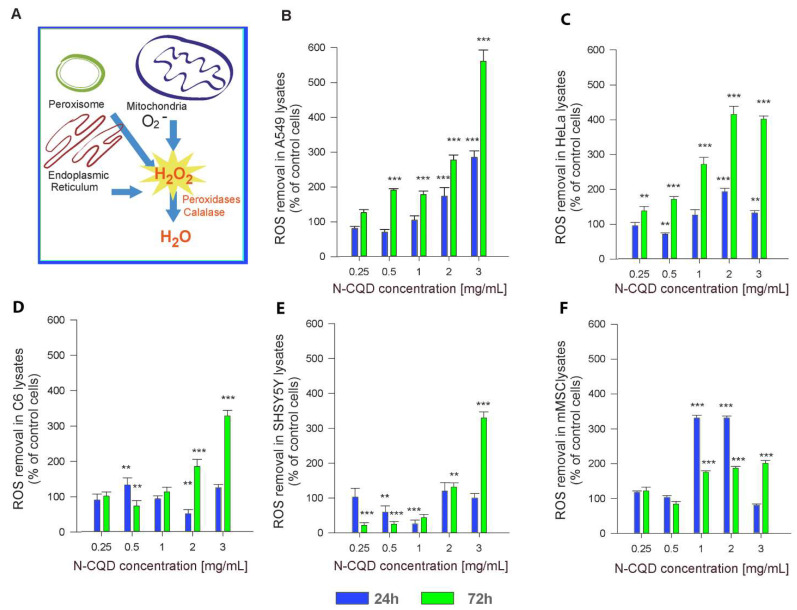
ROS removal ability in N-CQD-treated cell lysates with reference to control cells (without N-CQD): (**A**) schematic representation of ROS generation and removal inside the cells, (**B**) A549, (**C**) HeLa, (**D**) C6, (**E**) SH-SY5Y, (**F**) mMSC. All results are colored by culture time: 24 h (blue) and 72 h (green). The statistical significance of the differences is related to control samples and marked on the graphs with asterisks (* for *p* ≤ 0.05, ** for *p* ≤ 0.01, *** for *p* ≤ 0.001).

The potential resistance to N-CQD-derived ROS could be underpinned with an increased anti-oxidative potential of cells and their efficient defense against reactive oxygen species. The main sources of reactive oxygen species, as well as enzymes and pathways engaged in ROS removal inside the cell, are summarized schematically in Figure 4A. The imbalance between ROS generation and removal leads to oxidative stress. We have analyzed oxidative stress conditions by examining the cellular anti-oxidative capacity as a defensive response to the increasing concentration of N-CQDs. The ability to remove ROS was determined in cell lysates obtained after 24 h and 72 h treatment with 250, 500, 1000, 2000, and 3000 μg/mL N-CQDs, and related to the activity of control cells. It can be concluded that the carbonaceous nanomaterial in concentrations not exceeding 1000 μg/mL, despite being absorbed by cells, has not initiated oxidative stress conditions since no alterations in the anti-oxidative capacity have been observed in the majority of tested cells (Figure 4B–F). Only the most “responsive” cells –mMSC increased the anti-oxidative activity over 3 times after 24 h exposure to N-CQDs.

Summary of the above data leads to the conclusion about the non-toxic nature of CQDs. We postulate an explanation of the phenomena of N-CQD biocompatibility through their bio-interactions with proteins. The protein corona, which hinders the N-CQD oxidative properties, makes them cytocompatible and strongly justifies their application as therapeutic protein carriers favorably applied in cell systems.

The model enzymes: catalase, β-galactosidase, and apyrase were immobilized on N-CQD through physical adsorption and applying three different enzyme-to-carrier ratios allowed for controlled modulation of enzymatic activity similarly as it was described previously [14,32]. The adsorption phenomena are commonly known for inducing negligible or even no changes in the enzyme structure and functions. However, in the case of N-CQD support, immobilized enzymes exhibit changed kinetic parameters of the reaction, resulting in the modification of enzyme activity and efficiency compared to the activity of the native enzyme. What is more important, these changes are tunable, and the appropriate enzyme-to-carrier ratio allows for the customized modification of nanobiocatalytic systems.

Immobilized catalase (Table 2) reached the highest maximum velocity of the reaction, over 260 µmol/mL/min, at 1:10 enzyme to N-CQD ratio. This is a 30% increase in the V_max_ value regarding V_max_ of the native enzyme. However, it is associated with a higher K_m_ value, meaning the decreased catalytic efficiency, principally at low substrate concentrations. On the contrary, in the case of a 1.32:1 ratio, where the V_max_ is almost identical to the native catalase, the lowest K_m_ value of 1.522 mM has been determined, which results in an increase in the catalytic efficiency.

Immobilized β-D-galactosidase (Table 3) represents the class of hydrolases. Nevertheless, the observed changes in the kinetic parameters were similar to catalase at the N-CQD system and depended on the enzyme-to-nanomaterial ratio. The immobilization ratio of 1.32:1 resulted in a V_max_ almost identical compared to native enzyme, the lowest K_m_ value, and a considerable increase in the catalytic efficiency.

Immobilization of apyrase on N-CQD nanocarrier also triggered a positive modulation of enzyme catalytic properties (Table 4). The apyrase-to-nanomaterial ratio of 1.32:1, similarly as for the other enzymes, resulted in the highest substrate affinity together with substantially high activity. Correspondingly, the catalytic efficiency increased by 35% when compared with the native apyrase.

**Figure 5:**
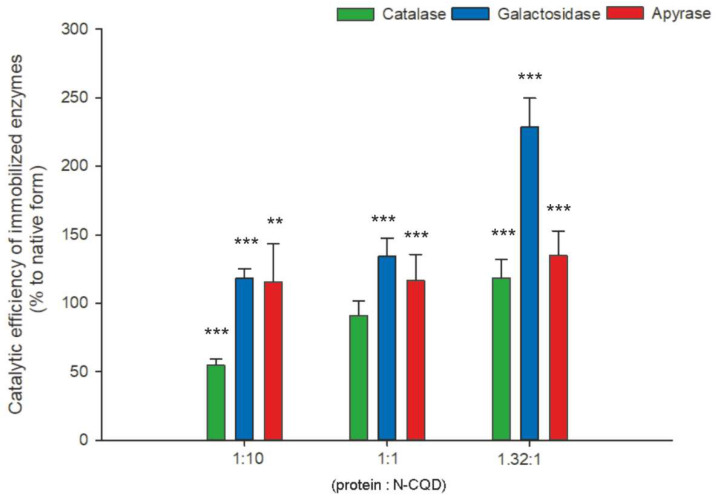
Comparison of catalytic efficiency of immobilized enzymes. The statistical significance of the differences is related to native enzymes and marked on the graphs with asterisks (* for *p* ≤ 0.05, ** for *p* ≤ 0.01, *** for *p* ≤ 0.001).

In general, the immobilization of enzymes on carbon quantum dots triggers the beneficial modulation of the catalytic efficiency of biocatalysts (Figure 5). The largest increase in this parameter, approximately two times in relation to the native enzyme, has been observed for β-D-galactosidase. However, it is worth noticing that the kinetic properties of all enzymes are tunable—immobilization starting from 1:1 protein to N-CQD ratio results in increased maximum velocity, whereas 1.32:1 ratio results in increased substrate affinity (lowered K_m_), which allows for controlled reactions at low substrate concentrations.

## 3. Discussion

Carbon quantum dots (CQDs), newcomers in the family of carbonaceous nanomaterials, are characterized by unique physicochemical properties, including their size with diameter range from approximately 1 to 20 nm, chemical passivity, excellent dispersibility in water, and facility for functionalization [1,25,33]. Additionally, intrinsic fluorescent properties, together with the relatively low toxicity of CQDs and ability to penetrate the cell membranes, enable them to be harmlessly implemented in cell and tissue bio-imaging, drug delivery, or in a combination of both diagnostic imaging and therapy, named theranostics [6,27,28,29,34]. For the last several years, the possibility of using nanomaterials as carriers of bioactive and/or therapeutic compounds has received increasing scientific interest; however, the use of carbon quantum dots as carriers for enzyme immobilization is rarely reported [28,35].

It is commonly accepted that CQDs may accumulate on the surface or inside cells, attach to proteins, cell membranes, or other biological structures, and generate increased oxidative stress, leading to cell damage or even death [1,3,36]. The ROS generation by N-CQDs has been determined through their ability to oxidize dichlorodihydrofluorescein, and was comparable with the millimolar concentrations of tBuOOH, commonly used as a radical initiator to induce free radical formation. To confirm N-CQDs’ cytocompatibility and phenomena of their lowered oxidative toxicity, we have focused on their interactions within the cellular microenvironment, specifically on the interplay with proteins. The protein corona formation of CQDs from roast salmon with serum albumin has only recently been reported and assumed to reduce the toxicity of quantum dots [37]. The bovine serum albumin coating strongly decreases their oxidative properties and does not quench their intrinsic fluorescence. Therefore, it ensures the N-CQD cytocompatibility and promotes their application, e.g., as therapeutic protein carriers for in vitro and in vivo experiments. It can be assumed that any other proteins, including enzymes immobilized on the N-CQD surface, will also have the beneficial effect of reducing oxidative properties and ROS production. Moreover, the enzyme immobilization will protect the pivotal intracellular proteins from being adsorbed and thus being unavailable for cells.

We tested the toxicity of N-CQDs at a wide range of concentrations and using five distinct cell lines. This allowed us to shed more light on the cytotoxicity issue. The existing literature data that have been reported differ considerably in cell lines used, concentrations, and incubation times [4,34,38,39]. It has been suggested by other researchers that carbon dots toxicity and cellular internalization may vary in different cell types, which is underpinned by various mechanisms of uptake and intracellular processing [7,8,39,40,41]. Based on our results, we can conclude that relatively high cell viability in the presence of N-CQD confirms that this kind of carbonaceous material can be safely used in various cell systems.

Carbon quantum dots are in general accepted to penetrate inside the cell and accumulate in its interior. Therefore, the amount of CQDs internalized by cells is of great importance in drug delivery systems. We have meticulously analyzed the fluorescence intensities of N-CQDs in the culture media collected after 24 and 72 h of culture, and based on this parameter, we were able to calculate the concentration of N-CQDs taken up by cells of each of the examined cell lines. Our results indicate that no components of the culture media changed the carbon quantum dots fluorescence intensity. Therefore, the culture media would not affect the possibility of the fluorescence-based determination of N-CQD concentration, which is contrary to the cytoplasm where N-CQD fluorescence can be quenched by reducing agents [30,31,42,43]. From the comprehensive results presented here, one can conclude that cells from examined cell lines differed in the rate of N-CQD internalization; however, the particular endocytic or other pathways engaged in nanomaterials internalization still need to be elucidated. HeLa and mMSCs, characterized with permeable extracellular membrane, take up and accumulate much more environmental components. The obtained results suggest that the rate of nanomaterial absorption by cells is essentially related to their membrane structure and phagocytic capacity. In HeLa cells, quantum dots have already been reported to be endocytosed by clathrin-mediated endocytosis and a small part of micropinocytosis, whereas in neural cells, carbon dots prepared by hydrothermal reaction underwent endocytosis via caveolae-mediated pathways [39,44,45]. The high nitrogen content in tested N-CQD ensures a cationic surface character that has been described to increase the internalization rate of particles [35], and experimentally confirmed by Gyulai et al. [41]. Based on our results, we postulate that the charged surface of N-CQD facilitates protein adsorption and the creation of protein corona.

The cell exposure to N-CQDs and nanomaterial uptake into cells did not trigger oxidative stress, damage or cell death. The potential resistance to N-CQD-derived ROS has also not been underpinned with the augmented anti-oxidative potential of cells. An increased capability for ROS removal after exposure to low N-CQD concentrations (up to 1 mg/mL) was observed only in mesenchymal stem cells, whereas high N-CQD concentrations increased ROS removal capability in A549 and HeLa cells considerably. Such activity can be explained by an intrinsic feature of cancer cells to produce an increased amount of ROS due to cytokine effects, active metabolism associated with continuous proliferation, and mutations in mitochondrial DNA [46,47].

According to the literature, the proper selection of the enzyme carrier for in vitro or in vivo applications should be based on fulfilling at least the conditions of a suitable immobilization surface, biocompatibility, and facilitated transport inside the cells [48,49]. The N-CQDs synthesized by our group meet these criteria. The use of carbon quantum dots as carriers for enzyme immobilization is rarely reported in the literature. Among carbon quantum dots, only graphene quantum dots were used to immobilize enzymes, benefiting enzymatic activity [50]. Enzyme immobilization is still claimed to acquire the prime position as a futuristic approach of high interest in the scientific community, industrial sectors, and biomedicine. It is equally important to select the ideal technique and support for enzyme immobilization, evaluate its interactions with the substrate and reaction kinetics [51]. However, the most recent literature data, suggesting that CQDs are promising nanodevices for theranostics, rarely deal with detailed biochemical analysis of immobilized molecules. Studies are needed to investigate the change of biochemical and biophysical properties in enzymes before and after they are in contact with nanoparticles. We expect our results to fill the gap in the evaluation of enzymatic processes in nanobiocatalysis, as the information about the interactions between nanoparticles and enzymes is still very limited [51,52,53].

The results of the presented study indicate that catalase, β-D-galactosidase and apyrase immobilized via physical absorption on the N-CQD surface increased their catalytic efficiency. Comparison of the kinetic parameters of native and immobilized enzymes shows alterations in the kinetics of the enzymatic reaction. Our results have confirmed that the protein to N-CQD ratio has decisive importance in modulating enzymatic activity, which was previously scarcely reported for other nanomaterials serving as immobilization support [14,54]. Depending on this parameter, an increase in the maximum reaction velocity or in the affinity of the enzyme towards substrate has been observed. Increased substrate affinity allows for controlled reactions in low substrate concentrations that are ubiquitous in physiological conditions in cells and tissues and is required for therapeutically important enzymes [55]. An excellent example can be apyrase, the enzyme that is responsible for the precise regulation of nucleotides balance in the extracellular environment. Thus, regarding the appropriate enzyme-to-N-CQD ratio, we can obtain the nanobiocatalytic system with kinetic parameters adjusted to the enzyme application.

## 4. Materials and Methods

### 4.1. N-CQD Preparation

The low-cost, green synthesis of nitrogen-containing carbon quantum dots via hydrothermal carbonization of gelatin was described previously by our group [30]. Briefly, 20 g gelatin was dissolved in 200 mL of deionized water. The solution was placed in a glass-lined Parr reactor for 6 h at 200 °C with a total pressure of 180 Bar. After cooling to room temperature, the material was used for experiments without further purification.

For the study of oxidative properties, the material has been coated with albumin: various concentrations of N-CQD were suspended in albumin solution (2 mg/mL) and incubated for 16 h.

### 4.2. In Vitro Cell Culture

Human epithelial carcinoma cells (HeLa) and rat glioma (C6) cell lines were obtained from Sigma-Aldrich (Darmstadt, Germany); mouse mesenchymal stem cells (mMSC) were purchased from Life Technologies (Poland); human lung epithelial cells A549 and human neuroblastoma cell line SH-SY5Y were purchased from ATCC collection. Cells were cultured in appropriate media (Ham’s F12 or DMEM) supplemented with 10% FBS (Biowest) under sterile conditions at 37 °C in a humidified atmosphere containing 4.9% CO_2_. After reaching subconfluency, the cells were trypsinized and approximately 2.5 × 10^4^ cells/cm^2^ were seeded in each well of 24-well plates for 24 h before adding N-CQDs. The appropriate concentrations of N-CQDs in culture medium ranging from 100 to 5000 μg/mL were added to the growing cells and cultured for 24, 48, and 72 h.

### 4.3. Viability Assays

The N-CQD influence on the cell proliferation and viability was assayed using the MTT (3-(4,5-dimethylthiazole-2-yl)-2,5-diphenyl tetrazolium bromide; Sigma-Aldrich, Darmstadt, Germany) test. The aliquots of 500 µL 1 mg/mL MTT solution in a suitable culture medium were added to each well. After 1 h of incubation at 37 °C, the solution was aspirated, 1 mL of dimethyl sulfoxide (DMSO; Sigma-Aldrich, Darmstadt, Germany) was added to each well and the plates were shaken for 10 min. The absorbance was measured at the wavelength of 570 nm with the subtraction of the 630 nm background, using a microplate reader. Cell viability has been calculated as the percentage of the sample to control absorbance.

### 4.4. Quantification of N-CQDs Internalization by Cells

N-CQDs solutions in the final concentration of 50 μg/mL were prepared in PBS buffer or different culture media with 10% FBS to assess the fluorescent properties in different solutions. The fluorescence was measured by a Tekan Spark Control reader at an excitation wavelength of 350 nm by registering the fluorescence spectra from 300 to 800 nm.

Evaluation of the N-CQD amount endocytosed by cells was performed after 72 h of culture in the presence of N-CQDs in 3 different initial concentrations (100, 250 and 500 μg/mL). The fluorescence intensity in media collected from the culture plates was measured at an excitation wavelength of 350 nm and emission at 415 nm. The amount of internalized material was calculated based on the differences between N-CQD fluorescence in control samples (without cells) and post-culture media and normalized to the number of cells.

### 4.5. Oxidative Properties of N-CQDs

Oxidative properties of N-CQD, pristine or coated with albumin, were detected through the material ability to oxidize 2′,7′-dichlorodihydrofluorescein (DCFH_2_) to fluorescent derivative with excitation maximum at 495 nm and emission at 530 nm according to [44]. Aliquots of 1 mM 2′,7′- dichlorodihydrofluorescein were added to the solutions of N-CQDs and tBuOOH and incubated at room temperature for 12 h. The fluorescence spectra were then recorded.

### 4.6. Determination of ROS Removal Capability

Anti-oxidative response of the cells was determined in cell lysates obtained after 24 and 72 h of cell incubation with N-CQDs. Cells were lysed using 250 µL lysis buffer with 1% Triton X-100. After centrifugation, lysates were collected. A measure of 2.9 mL of 25 mM hydrogen peroxide was added to 0.1 mL of lysate, mixed, and then the hydrogen peroxide amount was measured spectrophotometrically at 280 nm for 5 min. The decrease in absorbance reflected the substrate decomposition by antioxidant enzymes; the calculated ROS removal capability was normalized to the number of cells.

### 4.7. Enzymes Immobilization on Carbon Quantum Dots

Enzymes: recombinant β-D-galactosidase, EC 3.2.1.23 (Aflofarm, Poland), catalase, EC 1.11.1.6 (from bovine liver; Sigma-Aldrich), and recombinant apyrase, EC 3.6.1.5 (from Pichia pastoris; Sigma-Aldrich) were immobilized through physical adsorption on N-CQD. Stock solutions (0.25 mg of enzymatic protein in 1 mL) were prepared for all enzymes and mixed with N-CQD in different initial ratios of protein to nanomaterial concentration: 1:10, 1:1 and 1.32:1. The prepared mixtures were gently mixed and incubated under optimal adsorption conditions (24 h at 4 °C).

### 4.8. Determination of Catalase Activity

Determination of native and immobilized catalase activity was performed based on hydrogen peroxide decomposition. Shortly, 2.9 mL of hydrogen peroxide was added to 0.1 mL of appropriately diluted enzyme, mixed, and then the hydrogen peroxide amount was measured spectrophotometrically at 280 nm. For the determination of kinetic parameters (K_m_, V_max_), different substrate concentrations ranging from 0.5 mM to 25 mM were used.

### 4.9. Determination of β-D-Galactosidase Activity

For β-galactosidase activity determination, 0.1 mL of appropriately diluted enzyme was added to 0.2 mL p-nitrophenyl-β-d-galactopyranoside (NPG) in different concentrations ranging from 1.0 mM to 30 mM in 0.25 M phosphate buffer, pH = 6.0, containing 1 mM MgCl_2_ and 45 mM β-mercaptoethanol. After 5 min incubation, the enzymatic reaction was stopped by the addition of 0.5 mL 0.1 M NaOH. The absorbance of released p-nitrophenol was measured at λ = 405 nm.

### 4.10. Determination of Apyrase Activity

Apyrase activity was determined using a reaction mixture containing 50 mM Hepes buffer pH 6.5, 4 mM MgCl_2_, 2 mM CaCl_2_ and ATP as a substrate in different concentrations ranging from 0.5 mM to 5 mM. A measure of 20 μL of reaction mixture and 20 μL of the appropriately diluted enzyme were mixed and incubated for 20 min at 37 °C. The reaction was stopped by adding 40 μL of 1 M HClO_4_. The samples ware neutralized with 40 μL of 1 M KOH and centrifuged. Reaction products were quantitatively analyzed using HPLC system (Waters) on a Chromolith C-18 RP column (Merck) under isocratic conditions, in pH 7.0 phosphate buffer (0.1 M KH_2_PO_4_, 0.1 M K_2_HPO_4_, 5 mM EDTA, 25 mM TBA and 2.5% methanol). The presence of purines was detected at 260 nm.

### 4.11. Statistical Analyses

All experiments were performed in at least triplicate. The mean and standard deviation were calculated for the obtained values. Differences between the test groups were calculated with the Kruskal–Wallis test using PAST 4.02 software. The statistical significance of the differences is marked on the graphs with asterisks (* for *p* ≤ 0.05, ** for *p* ≤ 0.01, *** for *p* ≤ 0.001).

## 5. Conclusions

The nitrogen-containing carbon quantum dots described in this study present advances that go far beyond their cytocompatibility with different cell lines. N-CQDs are conveniently internalized by cells, and do not contribute to oxidative stress by reason of bio-interactions, mainly with intracellular proteins. When harnessed as a support in nanobiocatalytic systems, they are capable of improving the activity of immobilized enzymes and/or adjusting kinetic parameters to enzyme application. These properties make N-CQDs an ideal support for the immobilization of enzymes and other bioactive molecules. The phenomenon of tunable kinetics of biocatalytic process is essential for enzyme-based drugs. Therapeutic nanobiocatalytic systems are urgently required for numerous biomedical applications, including personalized medical therapies.

## Figures and Tables

**Table 1:** Concentration of N-CQD internalized by different cells within 72 h—calculations are based on the depletion of nanomaterial from culture media.

Cell Line	Depletion of N-CQD from Initial 500 µg/mL Concentration [%]	Concentration of Internalized N-CQD [ng per Cell]
A549	23.89 ± 0.5	1.91 ± 0.05
HeLa	59.38 ± 2.5	4.99 ± 0.4
C6	58.9 ± 1.2	3.34 ± 0.1
SH-SY5Y	45.2 ± 1.1	3.25 ± 0.1
mMSC	54.62 ± 3.1	3.79 ± 0.2

**Table 2:** Kinetic parameters of native and immobilized catalase. The statistical significance of the differences between native and immobilized enzyme is marked with asterisks (* for *p* ≤ 0.05, ** for *p* ≤ 0.01, *** for *p* ≤ 0.001).

Protein to N-CQD Ratio	V_max_ [µmol/mL/min] ± SD	K_m_ [mM] ± SD	V_max_/K_m_ [1/min]
Native enzyme	204.0 ± 13.1	1.78 ± 0.23	112.36
1:10 CQD	264.1 ± 25.4 **	4.22 ± 0. 65 ***	61.61
1:1 CQD	230.4 ± 20.8	2.35 ± 0.47 *	97.46
1.32:1 CQD	201.3 ± 11.8 **	1.52 ± 0.09 ***	131.58

**Table 3:** Kinetic parameters of native and immobilized β-D-galactosidase. The statistical significance of the differences between native and immobilized enzyme is marked with asterisks (*** for *p* ≤ 0.001).

**Protein to N-CQD Ratio**	**V_max_ [µmol/mL/min]** **± SD**	**K_m_ [mM]** **± SD**	**V_max_/K_m_** **[1/min]**
Native enzyme	17.27 ± 1.2	1.015 ± 0.09	17.01
1:10 CQD	17.30 ± 2.2 ***	0.861 ± 0.04 ***	20.08
1:1 CQD	20.16 ± 3.7 ***	0.881 ± 0.02 ***	22.88
1.32:1 CQD	17.57 ± 2.1 ***	0.451 ± 0.02 ***	38.91

**Table 4:** Kinetic parameters of native and immobilized apyrase. The statistical significance of the differences between native and immobilized enzyme is marked with asterisks (** for *p* ≤ 0.01, *** for *p* ≤ 0.001).

Protein to N-CQD Ratio	V_max_ [µmol/mL/min] ± SD	K_m_ [mM] ± SD	V_max_/K_m_ [1/min]
Native enzyme	4.207 ± 0.44	1.210 ± 0.04	3.478
1:10 CQD	3.827 ± 0.41 ***	0.952 ± 0.10 **	4.019
1:1 CQD	5.666 ± 0.38 ***	1.396 ± 0.09 ***	4.058
1.32:1 CQD	4.342 ± 0.43	0.925 ± 0.06 ***	4.693
